# Smoking Induces Long-Lasting Effects through a Monoamine-Oxidase Epigenetic Regulation

**DOI:** 10.1371/journal.pone.0007959

**Published:** 2009-11-23

**Authors:** Jean-Marie Launay, Muriel Del Pino, Gilles Chironi, Jacques Callebert, Katell Peoc'h, Jean-Louis Mégnien, Jacques Mallet, Alain Simon, Francine Rendu

**Affiliations:** 1 Service de Biochimie et Biologie Moléculaire/Equipe Associée (EA) 3621, Assistance Publique des Hôpitaux de Paris (AP-HP), Hôpital Lariboisière, Faculté de Pharmacie, Université Paris Descartes, Paris, France; 2 Signalisation cellulaire, dynamique circulatoire et athérosclérose précoce, Unité Mixte de Recherche (UMR) 7131, Université Pierre et Marie Curie (UPMC) Paris Universitas/Centre Nationale de la Recherche Scientifique (CNRS), Hôpital Broussais, Paris, France; 3 Centre de médecine préventive cardiovasculaire, AP-HP, Hôpital Européen Georges Pompidou-Broussais, Paris, France; 4 Laboratoire de Génétique Moléculaire de la Neurotransmission et des Processus Neurodégénératifs, UMR 7091, UPMC Paris Universitas/CNRS, Hôpital de la Pitié-Salpêtrière, Paris, France; 5 Faculte de Medecine Pitie-Salpetriere, UMRS 956 Inserm, INSERM, Paris, France; Institut Pasteur, France

## Abstract

**Background:**

Postulating that serotonin (5-HT), released from smoking-activated platelets could be involved in smoking-induced vascular modifications, we studied its catabolism in a series of 115 men distributed as current smokers (S), never smokers (NS) and former smokers (FS) who had stopped smoking for a mean of 13 years.

**Methodology/Principal Findings:**

5-HT, monoamine oxidase (MAO-B) activities and amounts were measured in platelets, and 5-hydroxyindolacetic acid (5-HIAA)—the 5-HT/MAO catabolite—in plasma samples. Both platelet 5-HT and plasma 5-HIAA levels were correlated with the 10-year cardiovascular Framingham relative risk (*P*<0.01), but these correlations became non-significant after adjustment for smoking status, underlining that the determining risk factor among those taken into account in the Framingham risk calculation was smoking. Surprisingly, the platelet 5-HT content was similar in S and NS but lower in FS with a parallel higher plasma level of 5-HIAA in FS. This was unforeseen since MAO-B activity was inhibited during smoking (*P*<0.00001). It was, however, consistent with a higher enzyme protein concentration found in S and FS than in NS (*P*<0.001). It thus appears that MAO inhibition during smoking was compensated by a higher synthesis. To investigate the persistent increase in MAO-B protein concentration, a study of the methylation of its gene promoter was undertaken in a small supplementary cohort of similar subjects. We found that the methylation frequency of the MAOB gene promoter was markedly lower (*P*<0.0001) for S and FS *vs.* NS due to cigarette smoke-induced increase of nucleic acid demethylase activity.

**Conclusions/Significance:**

This is one of the first reports that smoking induces an epigenetic modification. A better understanding of the epigenome may help to further elucidate the physiopathology and the development of new therapeutic approaches to tobacco addiction. The results could have a larger impact than cardiovascular damage, considering that MAO-dependent 5-HT catabolism is also involved in addiction, predisposition to cancer, behaviour and mental health.

## Introduction

Serotonin (5-hydroxytryptamine, 5-HT) is a potent biogenic amine, first described as a vasoconstrictor compound contained in the serum and later identified as a neurotransmitter [1]. Once synthesized in the gastrointestinal tract, peripheral 5-HT is actively taken up by platelets which store the amine within their dense granules. As a result, 5-HT is widely distributed in the body through the blood flow. 5-HT storage within platelets is held against such a high concentration gradient that it protects the organism from 5-HT-induced vascular tone abrupt modifications, maintains the 5-HT plasma level in the low nM range and prevents the amine of being degraded. Under normal physiological conditions, however, platelet granule-stored 5-HT can be either released into the blood flow through the open canalicular system or exposed to the platelet mitochondrial monoamine oxidase (MAO). In the latter case, it is degraded principally to 5-hydroxyindole acetic acid (5-HIAA) which also passes through the open canalicular system into the blood.

Degradation of bioamines mainly occurs through monoamine oxidases (MAOs). There are two MAO isoforms: MAO-A preferentially degrades endogenous bioamines such as 5-HT and norepinephrine, and MAO-B preferentially degrades exogenous bioamines such as phenylethylamine and benzylamine [2]. These specificities are relative, however. Human platelets and lymphocytes contain only the MAO-B isoform. Human *MAOA* and *MAOB* genes are both located on the short arm of the X chromosome (Xp.11.4–11.3, [3]). The two genes are arranged in a tail-to-tail orientation, and both span at least 60 kb, consist of 15 exons, and exhibit an identical exon-intron organization [4].

High 5-HT levels have been proposed to be predictive of coronary artery diseases, especially in young people [5]. In addition, inhibitors of 5-HT uptake, the so-called specific serotonin reuptake inhibitors (SSRIs) used in the treatment of depression and other psychiatric disorders [6], reduce the cardiovascular complications by inhibiting platelet activation and aggregation [7]. We therefore decided to measure the platelet 5-HT levels and to study its catabolism in a series of untreated healthy men at low risk for cardiovascular disease.

Unexpectedly, the greatest modifications in 5-HT catabolism were found in former smokers (FS) rather than in current smokers (S) as compared to subjects who had never smoked (NS). We found that smoking induced an epigenetic regulation of *MAOB*, *i.e.* a reduction of its gene promoter methylation, resulting in high MAO protein concentrations which persist long after (over 10 years) quitting smoking.

## Methods

### Subjects

The studied population was 115 men aged from 35 to 56 years free from stroke, transient ischemia, coronary heart disease, congestive heart failure and intermittent claudication. The usual biological variables were measured on an LX20 automate (Beckman-Coulter). Ten-year risk of coronary event was calculated by the Framingham equations on the basis of age, gender, systolic BP, total to HDL cholesterol ratio, and smoking [8]. Relative risk (RR) was calculated as the actual divided by the ideal (normotensive, normocholesterolemic, non smoker subject for each age category) 10-year coronary risk. All clinical investigations were conducted according to the Declaration of Helsinki principles and written informed consents were obtained from all subjects prior to inclusion in the study. The local Research Ethics Board approved the study protocols (Comité de Protection des Personnes Assistance Publique –Hôpitaux de Paris 06–223).

During the study it was decided to analyzed the methylation patterns of the *MAOB* core promoter. A supplementary cohort of 13 subjects were therefore recruited with consent for a genetic study.

### Smoking Status and Classification

Smoking status, duration of smoking, number of years since quitting and lifelong smoking dose were carefully assessed by questioning the subjects. Subjects were classified into three groups on the basis of their smoking status: (i) never smokers (NS); (ii) current smokers (S) – those who currently smoked daily or had smoked for the previous year, regardless of the amount smoked; and (iii) former smokers (FS) - those who had smoked, but had quit one year ago or more. Occasional smokers who had not quit for at least one year were not included in the study.

### Ultrasound Investigation

Ultrasonography was performed by experienced sonographer physicians using a 7.5 MHz probe for the extracranial carotid and femoral arteries and a 3-MHz probe for the abdominal aorta, as previously described [9]. Data were classified into two categories: absence or presence of any atherosclerotic arterial plaque(s). The intima media thickness (IMT) was measured on the common carotid artery on both sides in the far wall of at least 1 cm of longitudinal length, and calculated as previously described [10].

### Platelet, Peripheral Blood Mononuclear Cells (PBMC) and Plasma Preparation, Platelet Aggregation

Blood was drawn in 3.8% sodium citrate-anticoagulated tubes. Whole blood aggregometry was measured using 500 µL aliquots of blood diluted 1∶1 (v/v) in saline under constant stirring by the impedance technique, after addition of 2 µg/mL of collagen. Platelet rich plasma (PRP) was obtained by 10 min centrifugation at 100 g (20°C) and platelet poor plasma (PPP) by 10 min centrifugation at 20°C at 1500 g. Both were stored at −80°C until analysis (within 2 weeks). PBMC were obtained from each subject of the supplementary cohort using the standard Ficoll-Hypaque procedure.

### 5-HT and 5-HIAA Measurements

5-HT and its deaminated metabolite 5-HIAA were measured using HPLC, as described by Kema *et al.* [11] in PRP and PPP samples, respectively.

### MAO Activities and Protein Concentrations

MAO (EC.1.4.3.4.) enzymatic activity was determined as previously reported [12] on human PRP or PBMC samples by a radioenzymatic assay using [^14^C]-β-phenylethylamine (2.07 GBq/mmol, Amersham GE Healthcare, Saclay, F, final concentration 20 µM) as substrate. The MAO activity measured by this method is fully accounted for by MAO-B. Platelet MAO-B protein concentration was assessed for each subject by measuring the binding of [^3^H]-Ro 19-6327 [*N*-(2-aminoethyl)-5-chloropicolinamide HCl, lazabemide] (0.96 TBq/mmol, Amersham GE Healthcare), a reversible inhibitor of MAO-B, to human platelet membranes, as described by Cesura *et al.* [13] for [^3^H]Ro 16-6491. The same protocol was applied to human PBMC, mouse platelets and mouse lung tissue. MAO-A activities and concentrations in mouse lungs were determined exactly as for MAO-B except that [^14^C]-5-HT creatinine sulfate (1.96 GBq/mmol, Amersham GE Healthcare, Saclay, F,) and [^3^H]-Ro 41-1049 (0.31 TBq/mmol, Amersham GE Healthcare) were used as substrate and radioligand respectively. Platelet MAO-B protein concentration was also assessed by western blot: a total of 10 µg proteins were resolved by 4–12% Bis-Tris NuPAGE; separated proteins were electrotransferred to polyvinylidene difluoride membrane (Novex, San Diego, CA) and incubated with specific antibody to MAO-B (C-17, Santa Cruz Biotechnology Inc., Santa Cruz, CA).

### Sodium Bisulfite Genomic Sequencing of the *MAOB* Promoter

Genomic DNA was obtained from PBMC using standard procedures. Bisulfite treatments were performed as described [14] with minor modifications. A fragment of the genomic DNA of the *MAOB* promoter (Genbank M89637 5′flanking sequence −55 to −752 bp) was amplified using the following primers: 5′-GCCTTCCTGACTTAATCAC-3′ (forward -752) and 5′-CCTCGATCCCAGTCCTGCC-3′ (reverse -55). PCR amplification was performed with 5 µL of bisulfite-modified DNA with an annealing temperature of 56°C. PCR products were cloned into pCR II and sequenced.

### DNA Methyltransferase and Nucleic Acid Demethylase Activities of Mouse Lung

Male A/J mice, aged 6 to 8 weeks (Charles River, Orléans, F) were exposed to filtered air or mainstream cigarette smoke (10 mice in each group) using the exposure regimen developed by Witschi *et al.* [15] at a concentration of 250 mg/m^3^ of total suspended particulates for 5.5 hours/day, 5 days/week for 5 months, and sacrificed. All animal experimentation was performed in accordance with institutional guidelines (INSERM, Saint Louis Hospital) and approved by the French Animal Care Committee.

Nuclear extracts were prepared from lungs according to the protocol of Dignam *et al.* [16]. DNA methyltransferase activity was determined as described previously [17]. The nucleic acid demethylase activity of the mouse lung nuclear extracts was assayed as their ability to demethylate 3-methylcytosine in [^14^C]-methylated poly(dC). Demethylation was assayed according to [18], the only change being the replacement of the mouse reelin promoter by the mouse *MAOB* promoter.

### Statistical Analysis

It was carried out with the use of JMP (SAS) and Excel (Microsoft) softwares. For all tests, statistical significance was set at *P*<0.02 (α, two-sided type 1 error of <2%).

### Overall Study Sample

Continuous parameters are expressed as means ± SD. Normality of the distribution was analyzed by the Shapiro-Wilk W test. Normally-distributed variables were compared between smoking status groups by ANOVA, pairwise comparisons being performed by using the Student's t-test. Non normally-distributed variables were compared between smoking status groups by non parametric (Wilcoxon and Kruskal-Wallis) tests. Qualitative parameters are expressed as percent of subjects and compared between smoking status groups by the chi-square test. Linear regressions were used to analyze the relationships between continuous parameters, after logarithmic transformation in the case of non normal distribution.

In subjects recruited for the genetic study and in mice studies enzyme activities and concentrations were compared between groups by Fisher's exact test.

## Results

### Characteristics of Subjects

Subjects referred to the “Centre de Médecine Préventive Cardio-Vasculaire” between January 2003 and January 2007 for cardiovascular risk assessment were included in the study (i) if they had one or more mild cardiovascular risk factors among hypertension (defined as systolic blood pressure (BP) between 140 and 160 mm Hg and/or diastolic BP between 90 and 95 mm Hg), hypercholesterolemia (defined as total cholesterol after subjects had fasted for 14 hours between 5.2 and 7.2 mmol/L) and smoking; (ii) if they were free from diabetes (fasting blood glucose level <7 mmol/L), from obesity (body mass index <30 kg/m^2^) and from anti-hypertensive, lipid-lowering, anti-diabetic and platelet anti-aggregant therapies and SSRI drugs. The clinical profile of the studied population in relation to the smoking status is shown in Tables 1 and 2. The smoking group was typical [19], *i.e.* with more femoral plaques and higher number of atherosclerotic sites (Table 2). Smoking duration was higher in S than in FS group (*P*<0.02) but lifelong cumulative consumption, as reflected by pack.years, was not different between the two groups (Table 2).

**Table 1:** Baseline characteristics of 115 patients according to their smoking status.

	SMOKING STATUS			
Parameter	Non smokers (NS)	Smokers (S)	Former Smokers (FS)	*P* Value
	n = 34 (29.6%)	n = 44 (38.2%)	n = 37 (32.2%)	
**Age**, yrs	48±8	46±9	49±7	0.25
**Body mass index,** kg/m^2^	26±4	26±3	27±3	0.30
**Blood pressure,** mmHg				
systolic	127±15	126±12	129±15	0.71
diastolic	81±10	77±8	80±10	0.20
***Hypertensive patients,*** * n (%)*	*8 (23.5)*	*7 (16.0)*	*8 (23.5)*	*0.23*
**Lipid status**				
total cholesterol, mmol/L	5.5±0.7	6±1	5.7±1.1	0.10
HDL cholesterol, mmol/L	1.1±0.3	1.1±0.3	1.2±0.3	0.43
LDL cholesterol, mmol/L	3.6±0.9	4.1±0.9	3.7±1	0.03
***Hypercholesterolemic patients,*** * n (%)*	*24 (70.6)*	*33 (75.0)*	*26 (76.5)*	*0.87*
triglycerides, mmol/L	1.2±0.7	1.7±1.1	1.5±0.9	0.08
lipoprotein A, g/L	0.3±0.3	0.4±0.4	0.3±0.3	0.08
**Glycemia,** mmol/L	5.2±0.5	5.2±0.6	5.3±0.5	0.76
**Creatininemia,** µmol/L	87±11	81±12	85±10	0.52
**Folates,** ng/ml	6.5±3	6.3±3	5.6±2	0.41
**Homocystein,** µmol/L	9±2	10±3	10±3	0.24

Data are means ± SD or number and percent of subjects, n (%). There was no significant difference between NS, S, and FS and the frequencies of hypertension and hypercholesterolemia were similar in all three groups.

**Table 2:** Smoking, inflammation, blood parameters and atheromatous profile of 115 patients according to their smoking status.

	SMOKING STATUS			
Parameter	Non smokers (NS)	Smokers (S)	Former Smokers (FS)	*P* Value
	n = 34 (29.6%)	n = 44 (38.2%)	n = 37 (32.2%)	
**Smoking parameters**				(S *vs* FS)
cigarettes per day	0	19±3	0	-
yr of smoking	0	23±1	18±2	0.02
yr since quitting	0	0	13±8	-
packs.yrs	0	23±17	18±11	0.15
**Inflammation and blood profile**				(overall)
C reactive protein, mg/L	1.3±1.2	2±2	2±3	0.10
fibrinogen, g/L	2.8±0,5	3.1±0.5	3±0.4	0.06
hematocrit, %	42±2	44±3	43±2	0.06
hemoglobin, g/100 mL	14.4±0.7	14.8±0.8	15±1	0.10
leukocytes, G/L	5±1	7±2	6±1	0.0001
platelets, G/L	224±41	224±55	242±46	0.23
**Atheromatous profile**				(overall)
presence of plaque at any site, n (%)	14 (41)	31 (70.5)	22 (59.5)	0.05
presence of carotid plaque, n (%)	9 (26.5)	19 (43)	10 (27)	0.21
presence of femoral plaque, n (%)	9 (26.5)	29 (66)	16 (43)	0.002
presence of aortic plaque, n (%)	7 (21)	16 (36)	8 (22)	0.21
**Intima media thickness,** mm	0.56±0.07	0.60±0.10	0.60±0.10	0.55

Data are means ± SD, or number and percent of subjects n (%).

### Platelet Aggregation, Platelet 5-HT and Plasma 5-HIAA

**Figure 1:**
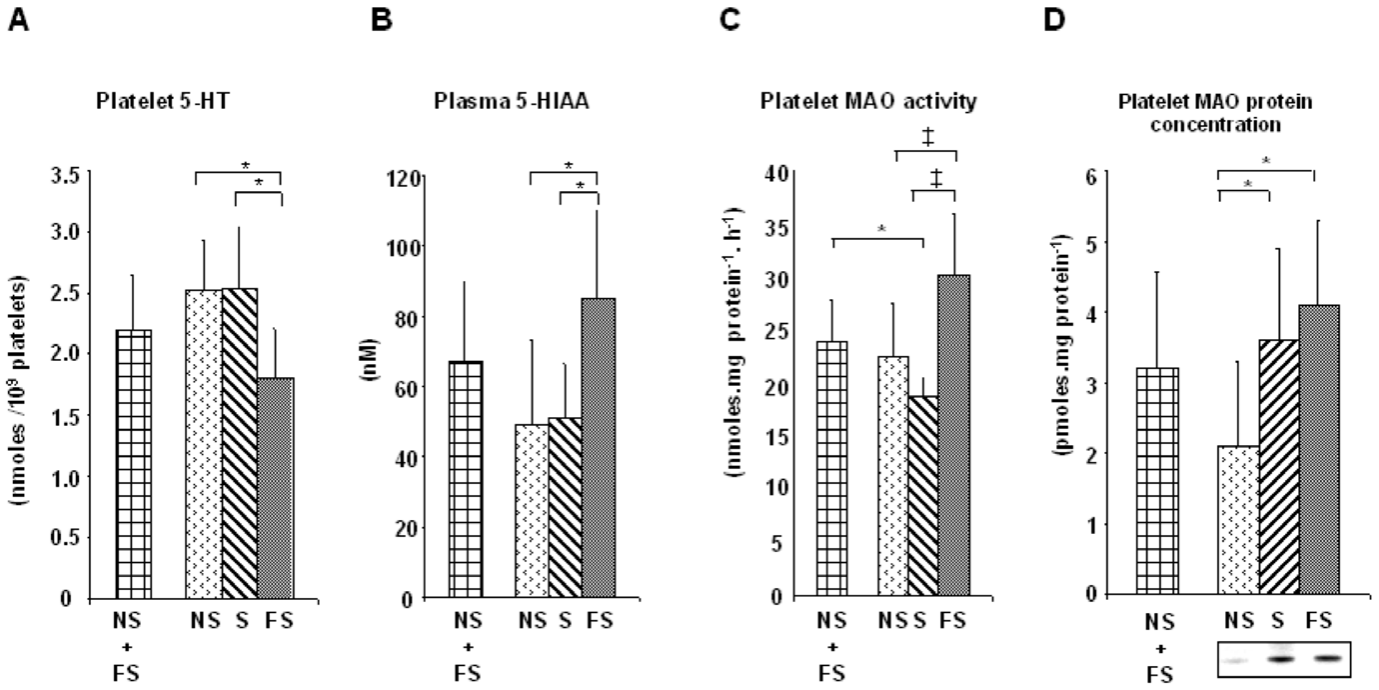
Platelet 5-HT catabolism according to smoking status. PRP 5-HT (1A) and PPP 5-HIAA (1B) were measured by HPLC in samples of 34 NS, 44 S and 37 FS. Results are means as nmoles 5-HT/10^9^ platelets and as nM 5-HIAA. Bars represent standard deviations. Significance: *P<0.001. None of the patients studied had a storage pool disease (<0.5 nmoles/10^9^ platelets). Platelet MAO-B activities (1C) were determined on PRP samples of 34 NS, 44 S and 37 FS by radioenzymology. Results are given as nmoles of substrate per mg protein and per hour. Platelet MAO-B protein concentrations (1D) were assessed both by Western blot and by measuring the binding of a reversible inhibitor of MAO-B to platelet membranes of 34 NS, 44 S and 37 FS and are expressed as pmoles of MAO-B per mg platelet protein. Results are mean values and bars represent standard deviations. Significance: *P<0.001; ‡*P*<0.00001.

Most studies dealing with smoking have compared S and NS, thus initially, in this study, only two groups were compared, *i.e.* current smokers [S] and current non-smokers who had not smoked for at least one year on the day of blood sampling (thus including never smokers [NS] and former smokers [FS]). In response to 2 µg/mL collagen, platelet aggregation amplitude in whole blood was similar in the two groups (16±5 *vs.* 16±4 ohms), whereas the aggregation velocity (ohms.min^−1^) was significantly lower (*P*<0.01) in S (7.9±0.5) than in NS+FS (10.3±0.6). The platelet 5-HT content was almost identical in the two groups (S and NS+FS), as was plasma 5-HIAA, (Fig. 1A, B).

This surprising lack of differences between current smokers (S) and current non-smokers (NS + FS) prompted a further analyze of the NS + FS group in order to differentiate those who had never smoked (NS) from those who had smoked but had quit one year ago or more, *i.e.* former smokers (FS). The less rapid aggregation of smokers' platelets was confirmed (velocity 7.9±0.5 ohms min^−1^ for S *vs.* 10.1±0.6 and 11.0±1.2 for FS and NS respectively, *P*<0.01). The distinction between NS and FS also indicated that platelet aggregation velocity returned to normal after quitting smoking.

The discrimination of FS in the non-smoking group demonstrated that FS had a significantly reduced platelet amount of 5-HT as compared to NS and S (Fig. 1A). It is therefore possible that 5-HT was released from the storage granules of FS platelets and, once released, was degraded to 5-HIAA. Indeed, the 5-HIAA amounts measured in FS plasmas were significantly higher than in NS and S (Fig. 1B).

### Platelet Monoamine Oxidase

The fact that platelet 5-HT was more degraded into 5-HIAA suggested that the enzyme responsible for this degradation, MAO, was either more active or more abundant in FS. MAO activity and MAO protein concentration were therefore measured in platelets since this is the most easily accessible source of MAO. As previously reported [2], [12], [20], MAO-B (the only isoenzyme present in human platelets) activity was significantly weaker (Fig. 1C) in current smokers (S) than in current non-smokers (NS + FS). But when discriminating FS in the current non-smoking group, MAO-B activity was found at a similar level in S and NS, whereas it was significantly greater in FS (Fig. 1C).

This higher platelet MAO activity found in FS was explained by a higher MAO-B protein concentration than in NS as assessed by both binding and western blot (Fig. 1D). Unexpectedly however, the platelet MAO protein concentration was also higher in S (Fig. 1D). Calculation, for each subject, of the specific activity of the enzyme, *i.e.* the ratio of MAO-B activity to its protein concentration, showed that the lowest mean MAO-B catalytic activity was that of S platelets where one enzyme molecule hydrolyzed 9,000 molecules of substrate per hour as compared to NS (13,000) and FS (11,000). Moreover, platelet 5-HT and plasma 5-HIAA levels were highly correlated (*P*<0.001, not shown) with both platelet MAO activities and protein concentration.

To our knowledge, this is the first report showing distinct measures of platelet MAO activities and protein concentrations in smokers. The present results reassert that MAO-B activity is inhibited by smoking and reveal for the first time that (**i**) platelet MAO-B protein concentration increased during smoking and (**ii**) this increase lasted long after quitting smoking (13 yrs average in our FS group, Table 2).

### 5-HT Catabolism and Cardiovascular Risk

**Figure 2:**
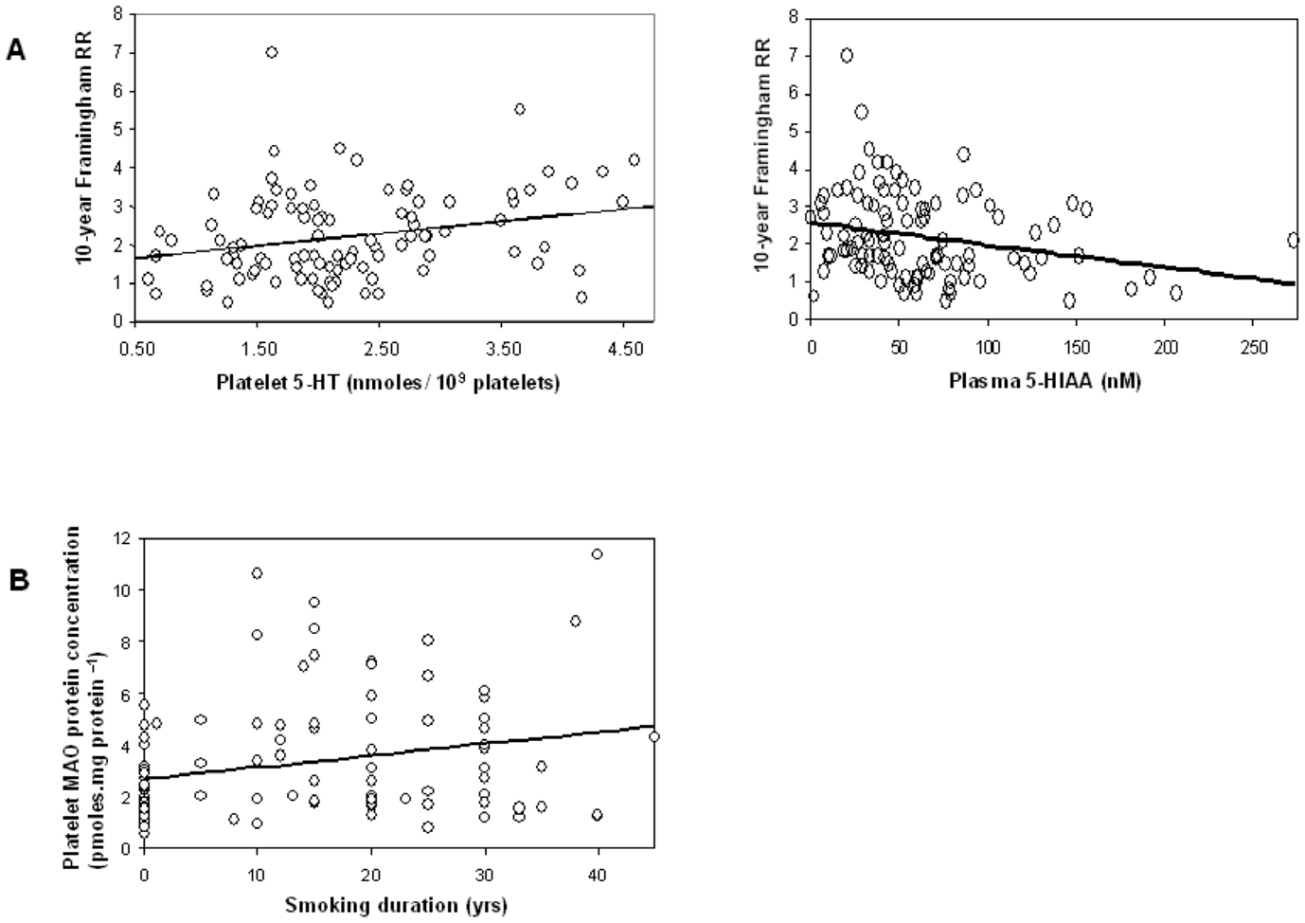
Serotonin catabolism, cardiovascular risk and smoking status. Correlations between (A) the Framingham Risk score and platelet 5-HT contents (n = 100, r = 0.27, *P*<0.01) or plasma 5-HIAA levels (n = 101, r = −0.25, *P*<0.02) and between (B) MAO-B amount and smoking duration (n = 101, r = 0.25, *P*<0.02).

In order to evaluate possible relationship(s) between the 5-HT catabolism and the cardiovascular risk, correlation(s) were sought between the studied markers of 5-HT catabolism and the cardiovascular risk reference, the Framingham risk score. Platelet 5-HT and plasma 5-HIAA levels did not correlate with smoking duration but were both significantly (*P*<0.02) correlated with the Framingham risk score, positively for platelet 5-HT and negatively for plasma 5-HIAA (Fig. 2A). However, these correlations became non significant after adjustment for smoking status, underlining that the determining risk factor among those taken into account in the Framingham risk calculation was smoking. In contrast, the platelet MAO protein concentration correlated (*P*<0.02) with the duration of smoking (Fig. 2B), but not with the Framingham risk score, indicating that the MAO protein concentration is linked to the life-long smoking rather than to the multifactorial cardiovascular risk. Finally, the MAO activity did not correlate with any studied marker other than platelet 5-HT or plasma 5-HIAA, except with the number of cigarette per day for S + FS (*P*<0.02, not shown) as previously observed [21].

### Methylation of the *MAO* Gene Promoter

The platelet MAO-B protein concentration remaining elevated several years after quitting smoking suggested a smoking-induced gene modification. A detailed analysis reported that the human *MAOB* core promoter contains 22 CpG sites that can be methylated, with methylation reducing the transcription of *MAOB* [22]. Such a genetic approach had not been considered when subjects were recruited. The methylation patterns of the *MAOB* core promoter were therefore analyzed in a supplementary cohort of subjects (5 S, 4 FS, 4 NS) with consent for a genetic study and clinically identical to the initial cohort (Table 3). This similarity also concerns metabolites related to methylation efficiency (*i.e.* folates and homocystein, *P*>0.05 between Tables 1 and 3 for these parameters). As for the previous population, the platelet MAO-B protein concentration (pmoles.mg protein^−1^) was the lowest in NS patients' platelets (1.1±0.1 *vs* 5.9±0.9 and 6.0±1.1 in S and FS, *P*<0.02), whereas the MAO-B activity (nmoles.mg protein^−1^.h^−1^) was the highest in FS platelets (57±15 *vs* 21±3 and 25±2 in S and NS, respectively; *P*<0.02).

**Table 3:** Baseline characteristics of the supplementary cohort recruited for the genetic study.

	Age (years of smoking)	Years since quitting	Total cholesterol (mmol/L)	HDL cholesterol (mmol/L)	LDL cholesterol (mmol/L)	Triglycerides (mmol/L)	Lipoprotein A (g/L)	Glycemia (mmol/L)	Créatininemia (µmol/L)	Folates (ng/ml)	Homocystein (µmol/L)
**NS**	45		5.4	1.1	3.1	1.4	0.3	5.4	91	7.6	9.1
**NS**	46		5.9	1.2	3.3	1.7	0.3	5.7	82	7.4	8.5
**NS**	43		4.8	1.2	3.5	1.3	0.4	5.2	89	7.5	10.4
**NS**	47		5.1	1.4	3.6	1.6	0.5	5.4	75	6.9	9.8
**FS**	50 (17)	9	5.5	1.4	4.9	1.4	0.3	5.1	91	8.9	9.2
**FS**	46 (16)	10	5.4	1.6	4.1	1.3	0.5	5.0	80	6.9	10.1
**FS**	47 (13)	13	5.3	1.3	4.4	1.3	0.5	6.3	81	8.1	11.0
**FS**	51 (12)	9	5.1	1.2	4.1	1.7	0.3	4.8	87	7.1	7.2
**S**	48 (19)		5.5	1.2	4.0	1.2	0.2	5.0	89	6.7	8.4
**S**	49 (17)		6.0	1.5	3.9	1.4	0.1	5.6	70	6.0	9.1
**S**	46 (15)		6.1	1.0	3.8	1.7	0.2	5.5	77	7.0	9.6
**S**	48 (16)		5.9	0.9	3.9	1.9	0.1	5.5	81	6.3	7.7
**S**	49 (14)		5.7	0.9	3.5	1.4	0.5	5.4	84	6.5	9.3

This new series was clinically identical to the initial cohort (Table 1).

**Figure 3:**
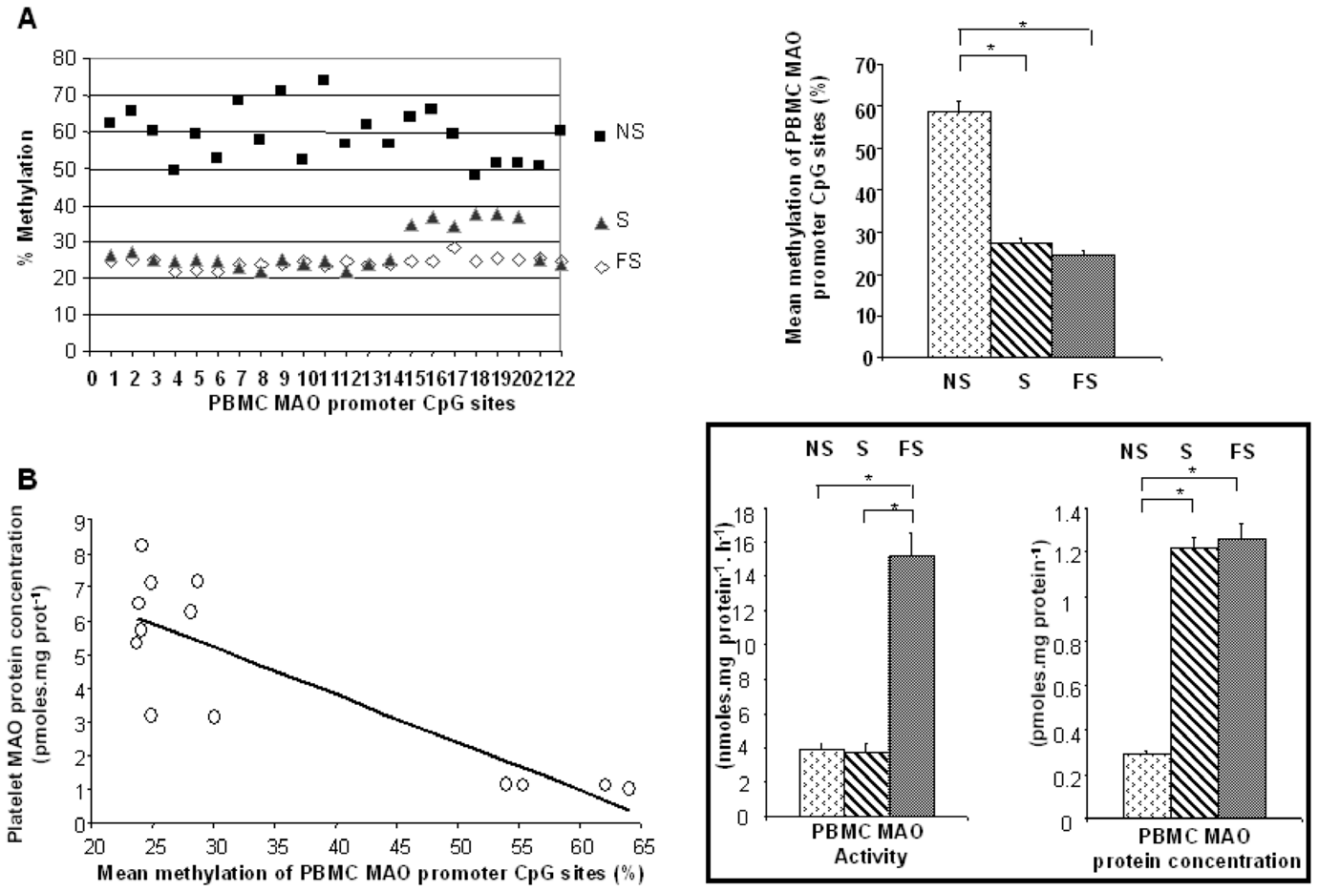
Analysis of the *MAOB* gene promoter methylation status. A Left: methylation frequencies at each CpG site within the human *MAOB* gene core promoter. Values (%) are means for each group (4 NS, 5 S, and 4 FS). A Right: mean methylation frequency of the 22 CpG sites of the *MAOB* gene promoter according to the smoking status (4 NS, 5 S, 4 FS) (*P<0.0001). B: correlation between the mean methylation frequencies of the *MAOB* gene promoter 22 CpG sites and the platelet MAO amounts (n = 13, r = 0.70, *P*<0.001). Insert: PBMC MAO-B activity (nmoles.mg protein^−1^.h^−1^) and protein concentrations (pmoles.mg protein^−1^) according to the smoking status of the subjects in the genetic study (4 NS, 5 S, 4 FS). Significance: *P<0.001.

DNA analysis was performed on PBMC of S, FS and NS of subjects of the supplementary cohort, which exhibited MAO profiles (Fig. 3, insert) similar to platelets. These confirmed that platelet MAO protein concentration was increased during smoking. The methylation frequency was notably higher for NS, both at each of the 22 sites (Fig. 3A, left) or as the mean of the 22 sites (Fig. 3A, right). The extent of methylation of each CpG in each sample is given in Table S1. According to the repressing effect of DNA methylation upon general gene transcription [23] and upon specific transcription of the human *MAOB* [22], the individual methylation frequencies were negatively correlated with the platelet MAO-B protein concentrations of the 13 studied patients (Fig. 3B). These findings offer an explanation for the high MAO-B protein concentration remaining long after quitting smoking.

**Figure 4:**
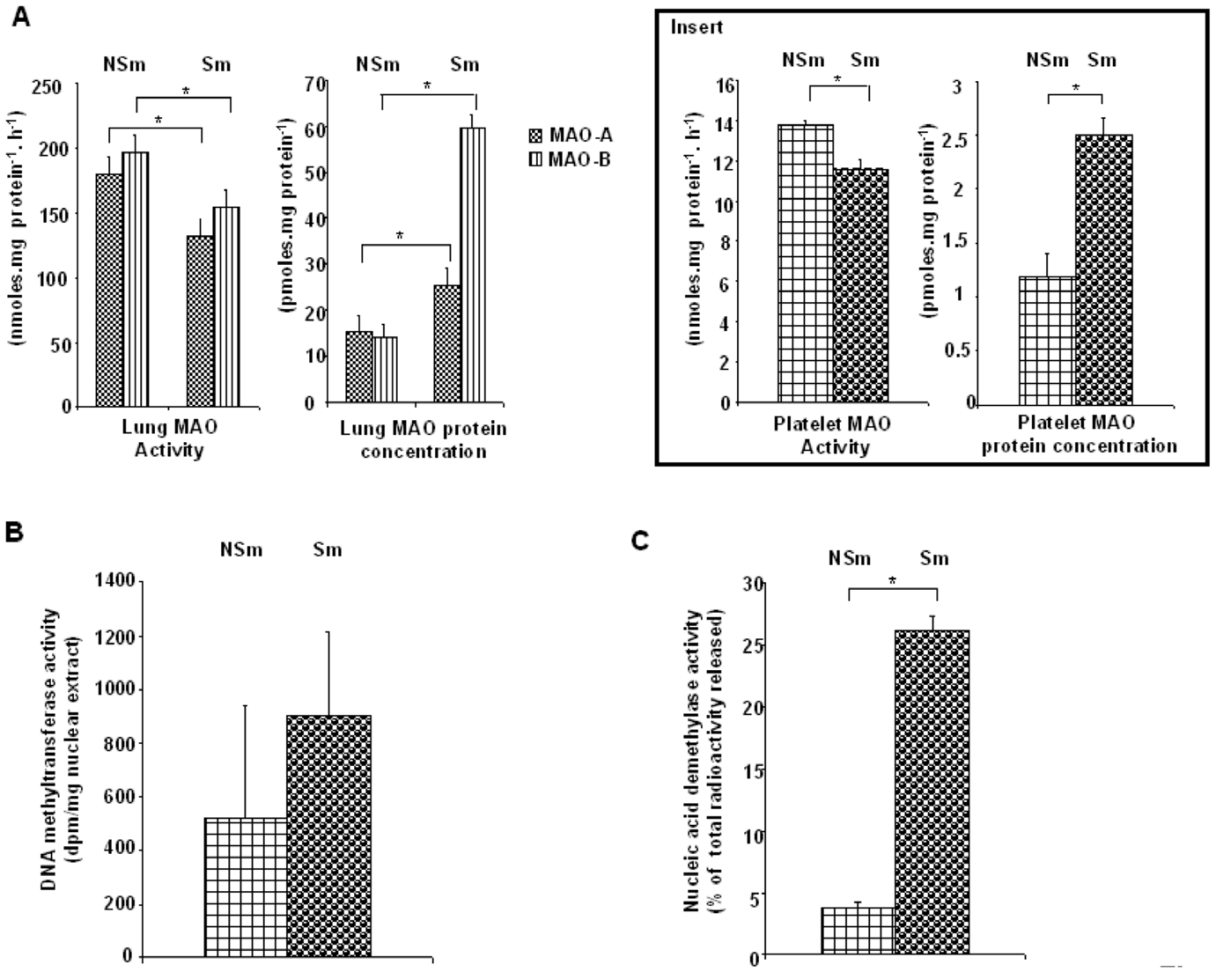
Effect of cigarette smoke on DNA methyltransferase and nucleic acid demethylase activities in mice. Mice (10 per group) were exposed (Sm) or not (NSm) to cigarette smoke and enzyme activities (A: MAO-A and MAO-B, B: DNA methyltransferase, and C: nucleic acid demethylase) determined on lung extracts as described in the methods. Results are expressed as means ± SD. Insert: platelet MAO activities (nmoles.mg protein^−1^.h^−1^) and protein concentrations (pmoles.mg protein^−1^) according to the smoking status of mice. Significance: *P<0.001

In order to verify that tobacco smoke is responsible for this methylation change, DNA methyltransferase and nucleic acid demethylase activities were measured in lung nuclear extracts of mice exposed to cigarette smoke. These mice exhibited platelet (MAO-B, Fig. 4 insert) and lung (MAO-B and MAO-A, Fig. 4A) MAO profiles comparable to those found in human S patient's platelets, *i.e.* higher protein concentration and lower activity in “smoking” mice (Sm) than in “non-smoking” mice (NSm). The PBMC methylation pattern of the *MAOB* core promoter was lower in Sm (27.6±3.4 *vs.* 48.7±2.5%, *P*<0.05). It is noteworthy that platelet 5-HT levels were increased after smoking (26.5±1.5 *vs.* 20.2±1.2 nmoles/mg protein, *P*<0.02) The lung DNA methyltransferase activity was not significantly different (Fig. 4B), whereas the nucleic acid demethylase activity was significantly higher in Sm (Fig. 4C). Moreover, the *in vitro* nucleic acid demethylase activity of mouse lung nuclear extracts was increased by harman and norharman, two known inhibitors of MAO present in tobacco smoke [24], [25]. Demethylase activity was increased by 12.3±0.4 and 9.6±0.5% for harman and norharman (100 nM), respectively (n = 3).

## Discussion

The present report is one of the very few to differentiate former smokers (FS) from those who have never smoked in terms of platelet 5-HT catabolism. 5-HT content was significantly reduced in platelets of FS who had quitted smoking for a mean of 13 years. This smoking-induced long-lasting effect was due to demethylation of the *MAOB* gene promoter which resulted in a persistent high protein concentration of platelet MAO-B. Moreover, results obtained in two species (human and mice), with three cell types (platelets, PBMC, lung cells) and concerning both MAO isoenzymes, indicate that this smoking-induced *MAO* gene deregulation could have a more general impact than vascular modifications.

Platelets from smokers have been reported to show increased aggregability [26] and to be more prone to spontaneous aggregation [27]. Platelet aggregation was studied with platelet rich plasma or isolated platelets. Under similar conditions, we could not find any difference (not shown), the sensitivity of the turbidimetric method possibly being too low. Platelet aggregation has also often been measured after acute smoking of 1–3 cigarettes, accounting for some discrepancies in the litterature. Here, we measured platelet aggregation in whole blood, *i.e.* in the physiological platelet environment which might differ from one group to the other. Fitting with a trend for a higher fibrinogen plasma level (Table 2) and thus a higher blood viscosity in S [28], the collagen-induced aggregation velocity was significantly lower for S than for NS and FS. This result emphasizes the importance of studying platelet functions in whole blood.

Platelet activation has been reported for both acute and chronic smokers with more activation markers exposed on platelet surface [29], [30]. The present results suggest that this smoking-induced platelet activation may not be sufficient to release the stored 5-HT from dense granules since similar 5-HT content was found in platelets from S or NS. Only FS platelets had lower levels of 5-HT, a finding consistent with the deficiency of the vesicular monoamine transporter reported in platelets of smokers [31] leading to a platelet dense granule 5-HT storage defect. Accordingly, we found a higher 5-HIAA level in FS plasma. The untransformed 5-HT released from platelet into plasma is largely diluted, and plasma 5-HT concentrations were not significantly different between the three groups (not shown).

Since there was no difference in other possible 5-HT metabolites (sulfo- and nitro(so)-conjugates, not shown), the higher level of 5-HIAA in FS plasma could result from either more active or in higher quantity of platelet MAO-B. MAO-B activity has been repeatedly reported to be lower in current smokers than in non smokers [2], [12], [20], [32]. Here, the distinct measurement of MAO activities and of MAO protein concentrations shows that the FS group accounts for the significantly higher platelet MAO-B activity found in “current non-smokers”. In addition, the platelet MAO protein concentration was elevated in FS and also in S. More MAO-B protein would be expected to result in higher MAO-B activity in the two groups. Yet, this was only true for FS but not for S. Calculation of the MAO-B catalytic activity, showed that the lowest activity was found in S platelets, in agreement with the low MAO-B activity typically found in current smokers [2], [12], [20]. This is consistent with the fact that MAOs are oxygen-dependent and carbon monoxide-inhibited enzymes [2]. This inhibition of MAO-B catalytic activity during smoking is also in agreement with PET-scan data obtained with brain [32] and peripheral organs [33] of smokers, non-smokers and ex-smokers using a radiotracer which binds irreversibly to MAO-B. On average MAO-B is inhibited by over 40% in smokers *vs.* non-smokers with no significant difference between brains of non- and ex-smokers. Two reports assessing whether MAO inhibition recovers after an overnight abstinence [34] or after 10–31 days of smoking abstinence [21] concluded that (**i**) the enzyme is irreversibly inhibited by compounds in cigarette smoke, and (**ii**) after smoking cessation, MAO activity probably returns to normal through *de novo* synthesis of the enzyme. The present study supports these conclusions and proposes a mechanism regulating MAO-B inhibition and slow recovery.

The present study shows that MAO activity was inhibited during smoking and restored after quitting, whereas MAO protein concentration was increased during smoking and remained high for many years after quitting. The persistence of increased protein concentrations of MAO-B was unexpected, even though some haematological and inflammation characteristics remain modified for several years after quitting smoking [19], [35], [36]. Smoking induced-modifications of the gene encoding MAO-B introduces a new explanation. Three recent studies identify an association between genetic variation on chromosome 15, in a region containing the gene encoding the nicotinic acetylcholine receptor, and risk of lung cancer [37]–[39]. A SNP is clearly linked to smoking intensity [38]. The present study demonstrates how epigenetics may also contribute to tobacco addiction and its long-lasting effects after quitting. A higher methylation of *MAOA* has been found associated with nicotine- and alcohol-dependence [40] in women whereas a down-regulation of *MAOA* transcription has been reported in several cancers including lung cancer [41]. Two recent studies reported molecular alterations in spontaneous sputum of cancer-free heavy smokers [42], [43] and an association of the offspring's global DNA methylation with paternal global DNA methylation, suggesting an association between smoking behaviour and global DNA methylation [44].

Both S and FS exhibited reduced methylation of the *MAOB* promoter, leading to a more active transcription of the gene and hence a greater protein concentration of MAO-B. Moreover, *ex vivo* animal experiments showed that tobacco smoke induced a reduction in the *MAOB* promoter methylation. This is the first report showing that smoking has a long-lasting effect in modifying *MAOB* transcription. To date modified *MAOB* transcription had only been shown during the differentiation of a human colon adenocarcinoma cell line [22]. DNA methylation is a covalent modification associated with long-term gene silencing with potential links to tumorigenesis [23] and suicide [45]. Thus, the epigenetic process found in the present report, which regulates gene activity without altering the DNA code, might explain at least some of the long-lasting effects and health problems generated by smoking [19], [35], [36].

**Figure 5:**
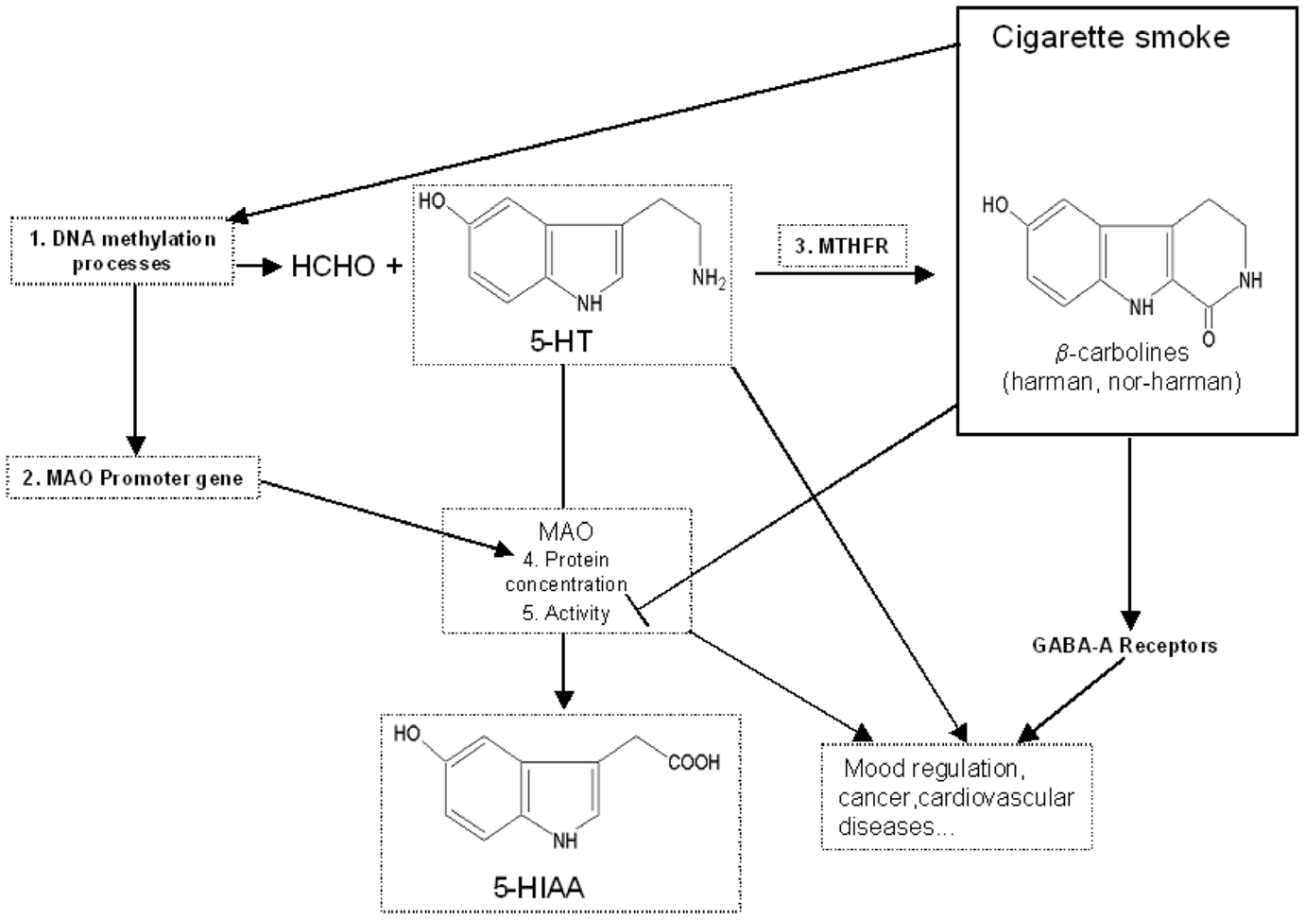
Effect of cigarette smoke on serotonin (5-HT) catabolism. Cigarette smoke stimulates the nucleic acid demethylase activity (1.) which generates aldehydes (HCHO) and demethylates the MAO promoter gene (2.). The demethylated MAO promoter gene leads to increased MAO protein concentration (4). The aldehydes, by their action on methylene tetrahydrofolate reductase (3. MTHFR), lead to the formation of β-carbolines, such as harman and norharman, from 5-HT. Harman and norharman are inhibitors of MAO activity (5.) and, being present in cigarette smoke*, keep the sequence 1–5 switched on in smokers. The β-carbolines also act on GABA-A receptors, thus contributing to mood regulation. *The *in vivo* levels of plasma harman (6.8 nM) and norharman (20.02 nM) reported in smokers [53] might also be high enough to stimulate nucleic acid demethylase activity, as it is the case for platelet MAO-B inhibition [53].

The nucleic acid demethylase activity, possibly due to hyperactive poly(ADP-ribose) polymerases [46], is increased by tobacco smoke (Fig. 4C) and generates aldehydes [24], [47] (Fig. 5) which are able to further cyclize bioamines to generate β-carbolines, such as harman or norharman [2], [24], [25] from 5-HT. In this way smoking might keep the MAO inhibited, contributing to a sustained 5-HT neurotransmission, thus mimicking an antidepressant effect (Fig. 5). In addition, β-carbolines, which are inverse agonists of GABA-A receptors, can play a role in the regulation of mood and anxiety states. Thus several data suggest that tobacco smoke may have mood regulating properties [2], [12].

In this context, epigenetic modifications have been reported in animal models of psychiatric disorders [48]. More specifically, keeping in mind that smoking is frequently abused by schizophrenia patients [49], nicotine (injected sub-cutaneously) was recently found to decrease DNA methyltransferase 1 expression and glutamic acid decarboxylase 67 promoter methylation in the mouse frontal cortex [50].

5-HT is at the crossroads of several cardiovascular and psychiatric diseases [51] and it has been suggested that MAO may play an important role at this cross-section, as already shown for alcoholic addiction [52] and predisposition to lung cancer [38], [41]. A better understanding of the MAO epigenome will help in further elucidating the physiopathology and developing new therapeutic approaches in tobacco addiction.

## Supporting Information

Table S1 Extent of methylation of each CpG in each patient sample (% of methylated clones)
(0.06 MB DOC)
