# Supplementary material for: Smoking Induces Long-Lasting Effects through a Monoamine-Oxidase Epigenetic Regulation
Source: PLoS One. 2009 Nov 23;4(11):e7959. doi: 10.1371/journal.pone.0007959 (PMC2775922; doi:10.1371/journal.pone.0007959)
Supplement: Table S1 — Extent of methylation of each CpG in each patient sample (% of methylated clones) (0.06 MB DOC) [file pone.0007959.s001.doc]

| **CpG site N°** | **1** | **2** | **3** | **4** | **5** | **6** | **7** | **8** | **9** | **10** | **11** | **12** | **13** | **14** | **15** | **16** | **17** | **18** | **19** | **20** | **21** | **22** | MM |
| --- | --- | --- | --- | --- | --- | --- | --- | --- | --- | --- | --- | --- | --- | --- | --- | --- | --- | --- | --- | --- | --- | --- | --- |
| **NS1** | 57.0 | 56.3 | 57.0 | 57.7 | 58.2 | 56.0 | 70.3 | 69.5 | 67.8 | 60.3 | 71.1 | 73.4 | 79.0 | 57.4 | 61.1 | 70.4 | 61.4 | 52.0 | 71.6 | 34.1 | 54.9 | 69.4 | 62.1 |
| **NS2** | 71.0 | 73.6 | 71.3 | 50.3 | 54.7 | 71.4 | 64.8 | 71.6 | 72.2 | 86.7 | 70.0 | 71.4 | 55.2 | 37.7 | 60.1 | 66.5 | 64.3 | 50.3 | 42.9 | 74.8 | 52.5 | 75.4 | 64.0 |
| **NS3** | 62.9 | 71.1 | 40.5 | 57.5 | 51.7 | 52.3 | 55.6 | 60.3 | 64.3 | 37.3 | 67.7 | 53.4 | 38.2 | 70.5 | 66.1 | 58.6 | 59.6 | 57.5 | 18.8 | 64.2 | 52.5 | 59.1 | 55.4 |
| **NS4** | 57.1 | 61.4 | 71.4 | 31.6 | 73.0 | 30.5 | 81.7 | 29.3 | 79.7 | 23.0 | 85.6 | 28.6 | 74.9 | 59.4 | 67.9 | 67.6 | 51.7 | 31.6 | 73.5 | 32.4 | 42.0 | 35.2 | 54.0 |
| **FS1** | 26.0 | 25.0 | 25.7 | 21.7 | 22.3 | 22.2 | 25.6 | 25.7 | 25.4 | 25.3 | 24.8 | 24.9 | 25.6 | 24.7 | 23.5 | 23.8 | 28.6 | 26.6 | 25.8 | 24.8 | 25.9 | 24.9 | 24.9 |
| **FS2** | 23.4 | 25.3 | 24.7 | 21.9 | 22.8 | 21.8 | 23.7 | 24.1 | 24.0 | 24.3 | 23.3 | 24.8 | 24.0 | 23.5 | 24.6 | 24.2 | 28.5 | 22.8 | 26.2 | 24.6 | 26.3 | 23.7 | 24.2 |
| **FS3** | 23.8 | 25.2 | 24.3 | 22.0 | 22.3 | 21.9 | 23.3 | 24.6 | 23.5 | 24.5 | 23.2 | 24.2 | 23.9 | 23.4 | 24.4 | 22.9 | 28.1 | 23.7 | 25.7 | 24.9 | 26.0 | 23.6 | 24.1 |
| **FS4** | 25.8 | 25.5 | 26.5 | 21.9 | 22.4 | 21.8 | 22.6 | 21.9 | 24.3 | 24.2 | 24.5 | 24.3 | 26.4 | 25.4 | 25.5 | 26.5 | 28.4 | 26.3 | 26.3 | 26.2 | 25.6 | 25.4 | 24.9 |
| **S1** | 24.2 | 24.6 | 24.6 | 23.1 | 22.3 | 21.7 | 22.4 | 21.3 | 23.9 | 24.3 | 22.9 | 23.1 | 23.0 | 24.8 | 24.6 | 24.2 | 26.7 | 24.4 | 25.2 | 24.7 | 24.5 | 24.6 | 23.9 |
| **S2** | 23.7 | 24.6 | 24.3 | 23.2 | 22.8 | 22.5 | 22.9 | 20.9 | 23.1 | 24.0 | 23.0 | 23.4 | 22.8 | 24.6 | 24.6 | 23.6 | 26.3 | 23.6 | 25.4 | 23.8 | 25.6 | 23.6 | 23.7 |
| **S3** | 28.1 | 27.8 | 27.7 | 27.4 | 26.3 | 26.0 | 22.2 | 21.8 | 27.0 | 24.2 | 27.4 | 24.5 | 24.8 | 25.5 | 40.7 | 41.6 | 41.2 | 43.4 | 40.8 | 44.4 | 25.5 | 24.9 | 30.1 |
| **S4** | 26.1 | 28.3 | 23.6 | 23.5 | 26.0 | 25.1 | 22.6 | 22.9 | 25.3 | 23.6 | 24.6 | 21.1 | 24.5 | 24.8 | 39.6 | 40.7 | 35.1 | 41.6 | 42.4 | 41.7 | 25.6 | 24.5 | 28.8 |
| **S5** | 26.3 | 28.4 | 23.4 | 23.4 | 26.1 | 24.9 | 23.6 | 22.2 | 25.2 | 23.6 | 23.6 | 20.9 | 23.6 | 24.7 | 34.1 | 40.8 | 34.1 | 42.4 | 41.8 | 38.6 | 24.9 | 23.8 | 28.2 |

**Supplementary table S1 - Extent of methylation of each CpG in each patient sample**

(% of methylated clones)**;** MM : mean methylation
